# Do We Need to Use a Stent in Biliary Reconstruction to Decrease the Incidence of Biliary Complications in Liver Transplantation? A Systematic Review and Meta-Analysis

**DOI:** 10.1007/s11605-022-05479-7

**Published:** 2022-11-14

**Authors:** Beshoy Effat Elkomos, Amr Abdelaal

**Affiliations:** General Surgery Department, Ain Shams University Hospital, Cairo, Egypt

**Keywords:** Liver transplant, Stent, Biliary complications

## Abstract

**Background and Aim:**

Biliary complications are a significant cause of morbidity post-transplantation, and the routine use of biliary stents in liver transplantation to reduce these complications remains controversial. This study aimed to compare the incidence of biliary complications with and without the use of trans anastomotic biliary stent in liver transplantation.

**Method:**

PubMed, Scopes, Web of Science, and Cochrane library were searched for eligible studies from inception to February 2022, and a systematic review and meta-analysis were done to compare the incidence of biliary complications in the two groups.

**Results:**

Seventeen studies with a total of 2623 patients were included. The pooled results from the included studies showed an equal rate of biliary complications (i.e., strictures, leaks and cholangitis) in stented and non-stented patients after liver transplantation. However, the cost and biliary intervention rates are higher in stented patients. In addition to that, our sub-group analysis showed no significant decrease in the incidence of biliary complications after using trans anastomotic biliary stent in living donor liver transplant (LDLT), deceased donor liver transplant (DDLT), Roux-en-Y hepaticojejunostomy (RYHJ), and duct-to-duct anastomosis, pediatric, and adult liver transplantation.

**Conclusion:**

No added benefit on the routine use of endobiliary stent in liver transplantation. However, stented patients are at higher risk of needing multiple ERCPs.

Liver transplantation (LT) has become a standard of care for acute liver cell failure, end-stage liver disease, and primary liver cancer with liver cirrhosis.^1^ Biliary complications remain the leading cause of mortality and morbidity after liver transplantation. Their overall rate ranges from 15 to 25%. With associated mortality of 10%.^2^ These complications include strictures, bile leakage, and cholangitis.^3^ Bile duct complications often depend on the type of transplant graft, either a living donor or deceased donor liver transplant, the number of bile ducts involved in the anastomosis, and the anastomosis itself used by the surgeon (choledocho-choledochotomy or hepaticojejunostomy).^4^

Despite using biliary stent by most transplant centers as a way to decrease the incidence of biliary complications, there is limited evidence in the literature for its necessity in biliary anastomosis. On the one hand, some studies reported that the use of biliary stents appeared to dramatically decrease the rate of biliary complications.^5,6^ On the other hand, other studies found that trans-anastomotic biliary stent was not required in liver transplantation to decrease the incidence of biliary complications and biliary reconstruction without stenting was a safe and efficient technique.^7,8^ In addition to that, as reported by some studies, the routine use of endobiliary stents is associated with a remarkable increase in the biliary complications and increases the rate of biliary intervention. This study aimed to compare the rate of biliary complication with and without the use of trans anastomotic biliary stent (internal and external) in liver transplantation. Moreover, we aimed to detect the outcome of using stent in different modalities of liver transplantation (LDLT and DDLT), different age groups (adult and pediatric liver transplantation), and various types of reconstructions (duct to duct anastomosis and hepaticojejunostomy).

## Patients and Methods

### Search Strategy

The search was directed through PubMed/MEDLINE, Scopus, Web of science, and the Cochrane Library for information from inception to February 2022 with a combination of the following terms: Liver transplantation, stent, and biliary tract. All studies were reviewed and evaluated by two authors (Elkomos, B. E. and Abdelaal A. A.). according to the eligibility process. Abstract-based eligibility studies were obtained, and the manuscripts were fully reviewed.

### Inclusion and Exclusion Criteria

The eligible studies included the following: (1) randomized controlled trials and prospective or retrospective cohort studies; (2) target populations were all patients underwent liver transplantation; (3) studies designed to compare the outcomes of biliary anastomosis using stent versus no stent; (4) studies providing a sufficient description of the methods and baseline characteristics; and (5) the primary outcome was postoperative complications including cholangitis, stricture, and leakage for the two groups. The following types of studies were not included in our study: (1) unrelated or in vitro studies; (2) reviews, case reports, and case series; and (3) studies missing a comparison group.

### Outcomes of Interest

We assessed the postoperative complications for using stent in biliary anastomosis versus biliary reconstruction without using stent in liver transplantation, including biliary stricture, cholangitis, and leakage. In addition to that, our aims were to detect the effect of using various types of stents (internal and external stent). Our sub-group analysis aimed to detect the outcome of using stent in different modalities of liver transplantation (LDLT and DDLT), different age groups (adult and pediatric liver transplantation), and various types of reconstructions (duct to duct anastomosis and hepaticojejunostomy).

#### Data Extraction

We extracted data on study characteristics (author, year of publication, country of transplant, study design and study period), patient characteristics (sample size, follow-up period, causes of transplantation, and Child score), operative details (type of graft, type of biliary anastomosis, and type of stent used), and study outcomes (incidence of postoperative cholangitis, stricture, and leakage for stented and non-stented group). The data were extracted by 2 investigators (Elkomos, B. E. and Abdelaal A. A.) independently.

#### Statistical Analysis

The meta-analysis was conducted according to Cochrane Handbook for Systematic Reviews of Interventions,^9^ which is recommended by the Cochrane Collaboration. Regarding postoperative complications (stricture, cholangitis and leakage), the pooled risk ratios (RRs) and their corresponding 95% confidence intervals (CIs) were calculated with fixed effects models. However, if there was moderate or considerable heterogeneity (*I*^2^ > 40), random-effects models were used to solve the heterogeneity between studies. All calculations for the current meta-analysis were performed with Review Manager 5.4 for Windows (Cochrane Collaboration, Oxford, UK).

#### Assessment of Publication Bias and Heterogeneity

Funnel plots were generated so that we could visually inspect for publication bias. The statistical methods for detecting funnel plot asymmetry were the Begg Mazumdar rank correlation test and the Egger regression asymmetry test. Statistical heterogeneity was assessed with forest plots and the inconsistency statistic (I2). An *I*^2^ value of 40% or less corresponded to low heterogeneity. Statistical significance was considered at *P* < 0.05.

## Results

### Characteristics and Quality Assessment of Eligible Studies

**Fig. 1:**
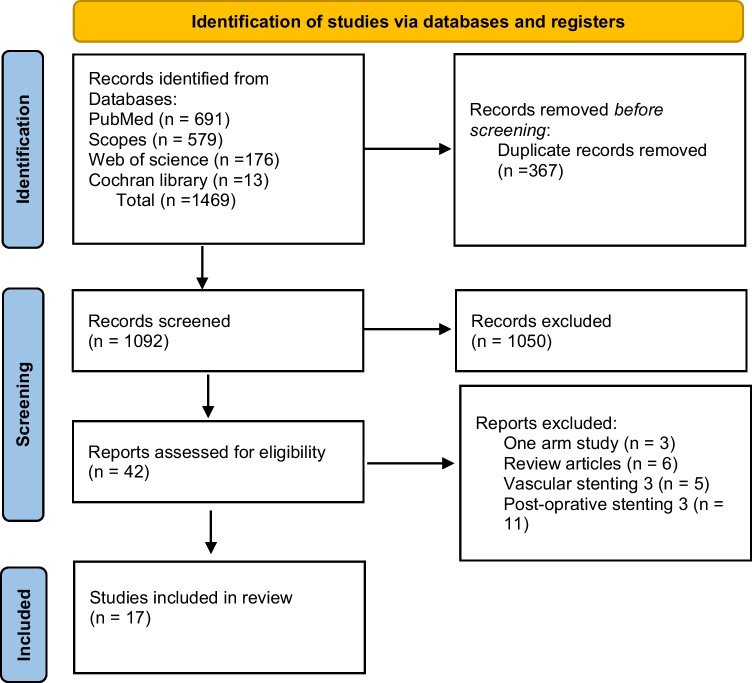
PRISMA flow diagram

As shown in the flow diagram (Fig. 1), 1469 articles were detected using the following search string: liver transplantation, stent, and biliary complications. According to our eligibility criteria and after careful selection, 17 studies^8,10–25^ with total of 2623 patients were included in the meta-analysis. These trials included 13 retrospective cohort studies^8,10,11,13–21,24,25^ and 4 randomized controlled trials.^12,]^^21–23^ All of them were single center studies. Eight studies used internal stent, seven studies used external stent, and two studies used both types. While adult liver transplantation was conducted in 13 studies, only 4 studies reported pediatric liver transplantation.

Recipients baseline data [including number, age, sex, and waiting time], follow up time, and the tumor-related baseline variables [including percentage of patients beyond the Milan or UCSF criteria, number of tumors, tumor differentiation, the size of largest tumor, microvascular invasion, macrovascular invasion, Model for End-Stage Liver Disease (MELD) score, Child–Pugh class, and treatment before LT] were comparable between groups in all studies (Table 1).

**Table 1:** Basic data of the included studies

Studies: author, year, country	Study design	Study period	No. of centers	Arm	Sample size (*n*)	Type of anastomosis (DD/HJ)	Type of graft Whole liver/ left lobe / right lobe / living donor	Age (yr)	Gender: Male/ Female(*n*)	Follow-up period (months)	Indication of transplant:End stage liver disease/ malignant	Child-Pugh Class: A/B/C(*n*)
Bawa, S. M. (1998) UK^12^	RCT	1995-1996	1	stent (internal)	18	18/0	N/A	53 (13-66)	N/A	19 (13-26)	N/A	N/A
				No stent	19	19/0	N/A	49 (19-69)	N/A	19 (13-26)	N/A	N/A
Egawa, H. (2001) Japan^13^	Retrospective study	1990-1998	1	stent(Internal/external)	135 (59/76)	1/134	LDLT	ped	N/A	N/A	N/A	N/A
				No stent	258	0/242	LDLT	ped	N/A	N/A	N/A	N/A
Kasahara, M. (2006) Japan^14^	Retrospective study	1998-2004	1	stent(Internal/external)	296(30/266)	N/A	296 rt lobe LDLT	N/A	N/A	N/A	N/A	N/A
				No stent	19	N/A	19 rt lobe LDLT	N/A	N/A	N/A	N/A	N/A
Haberal, M. (2006) Turkey^15^	Retrospective study	2001-2006	1	stent (external)	53	42/11	N/A	N/A	N/A	N/A	N/A	N/A
				No stent	13	10//3	N/A	N/A	N/A	N/A	N/A	N/A
Kobayashi, T. (2007) Japan^16^	Retrospective study	1999-2004	1	stent (External/T-tube)	11//6	0/17	0/0/17/0	N/A	7//10	N/A	5//12	N/A
				No stent	11	0/11	0/0/11/0	54(17-63)	4//7	31 (8–64)	3//8	2//3//6
Sakamoto, S. (2008) Japan ^17^	Retrospective study	2000-2006	1	stent (external)	14	14/0	LDLT	ped	N/A	N/A	N/A	N/A
				No stent	5	5//0	LDLT	ped	N/A	N/A	N/A	N/A
Spada, M. (2009) Italy^18^	Retrospective study	N/A	1	stent (external)	7	0/7	DDLT	ped	N/A	N/A	N/A	N/A
				No stent	68	7/61	DDLT	ped	N/A	N/A	N/A	N/A
Wang, GS (2013) China^19^	Retrospective study	2003-2009	1	stent (external)	25	0/25	N/A	49.0±13.9	21//4	646	16//9	N/A
				No stent	27	0/27	N/A	46.3±12.5	25//2	649	16//11	N/A
Jung, S. W. (2014) Korea^20^	Retrospective study	2009-2013	1	stent (internal)	31	30//1	13/2/16/-	52 ± 10.09	20/11	N/A	9//22	6/10//15
				No stent	17	16//1	15/1/1/-	51.65 ± 9.19	9//8	N/A	11//6	2/2//13
Mathur, A. K. (2015) USA^9^	Retrospective study	2006-2012	1	stent (internal)	221	215/6	N/A	52.6 ± 9.7	154/67	N/A	150/71	N/A
				No stent	292	233/59	N/A	52.3 ± 10.3	111/181	N/A	214/78	N/A
Janousek, L. (2016) Czech^21^	RCT	N/A	1	stent (biodegradable internal)	5	5//0	N/A	58.5±15	2//3	N/A	N/A	6.5±1.7
				No stent	5	5//0	N/A	59.5±7.8	3//2	N/A	N/A	9.5±1.4
Valentino, P. L. (2016) USA^10^	Retrospective study	2005-2014	1	stent (external)	56	14/42	26/28/4//3	1.7 (0.6, 7.6)	26//30	5.1 (3.6, 6.8) YS	46//10	N/A
				No stent	43	18/25	22/13/8/7	2.2 (0.7, 10.0)	19//24	1.1 (0.6, 2.0) YS	35//8	N/A
Kaneko, J. (2016) Japan^22^	RCT	N/A	1	stent (external)	63	N/A	LDLT	Adult	N/A	N/A	N/A	N/A
				No stent	59	N/A	LDLT	Adult	N/A	N/A	N/A	N/A
Kumar, K. S. (2017) India^23^	RCT	2013-2015	1	stent (internal)	31	N/A	31 LDLT	43.4 ± 11.1	N/A	N/A	N/A	N/A
				No stent	33	N/A	33 LDLT	48.5 ± 11.6	N/A	N/A	N/A	N/A
Ong, M. (2018) Australia^8^	Retrospective study	2011-2015	1	stent (internal)	88	88/0	N/A	55 (49–59)	64/23	N/A	62/26	N/A
				No stent	37	37/0	N/A	51 (45–57)	28/10	N/A	28/9	N/A
Girard, E. (2018) France^24^	Retrospective study	2009-2013	1	stent (internal)	67	67/0	67/0/0/0	56 (49–60)	58/9	N/A	49/18	N/A
				No stent	108	108/0	108/0/0/0	57 (50–62)	86/22	N/A	61/47	N/A
Yoon, Y. C (2021) USA^25^	Retrospective study	2008-2018	1	stent (internal)	132	132//0	N/A	57 (45-65)	89/43	N/A	N/A	N/A
				No stent	350	350/0	N/A	55 (43-64)	229/121	N/A	N/A	N/A

^*^Results are presented as means and standard deviation

^**^The results are presented as median and range

### Overall Complications

**Fig. 2:**
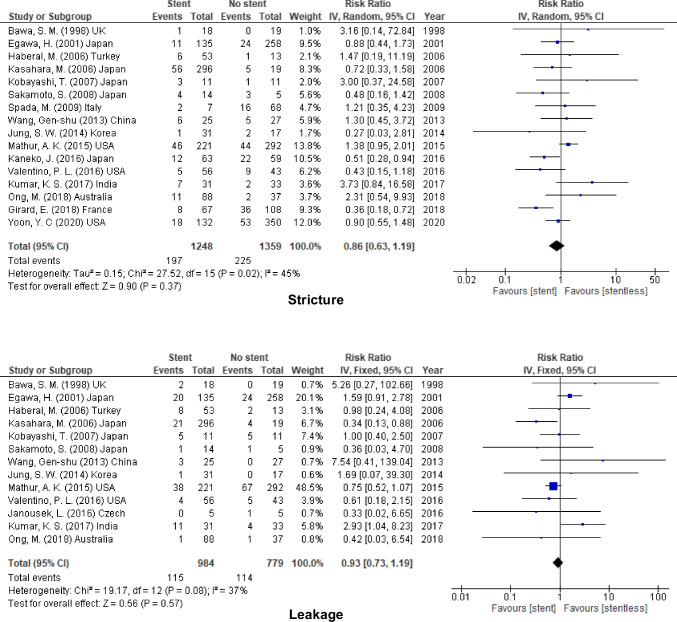
Overall incidence of biliary complication for stent (internal or external) vs. stentless

Sixteen studies (2607 participants) assessed overall postoperative stricture after using biliary stent (internal or external) versus biliary anastomosis without stent, 13 studies (1763) calculated the overall incidence of leakage, and 5 studies (794) calculated the overall rate of leakage for the two groups. The pooled results from these studies no significant difference in the incidence of postoperative biliary complications between the two groups (stricture, RR = 0.86, 95% CI = 0.63–1.19, *P* = 0.37; *I*^2^ = 45%), (leakage, RR = 0.93, 95% CI = 0.73–1.19, *P* = 0.57; *I*^2^ = 37%) and (cholangitis, RR = 1.32, 95% CI = 0.95–1.83, *P* = 0.10; *I*^2^ = 0%) (Fig. 2; Table 2).

**Table 2:** The outcome of using stent (internal & external stent) in different modalities

Outcomes			Studies (*n*)	Patients (*n*)	Effect Estimate [RR (95% CI)]	Heterogeneity	Test for Overall Effect	Favour group
Overall incidence		Stricture	16	2607	0.86 [0.63, 1.19]	I^2^ = 45% (*P* = 0.02)	Z = 0.90 (*P* = 0.37)	None
		Leakage	13	1763	0.93 [0.73, 1.19]	I^2^ = 37% (*P* = 0.08)	Z = 0.56 (*P* = 0.57)	
		Cholangitis	55	794	1.32 [0.95, 1.83]	I^2^ = 0% (*P* = 0.76)	Z = 1.64 (*P* = 0.10)	
Internal stent		Stricture	9	1810	1.03 [0.66, 1.60]	I^2^ = 54% (*P* = 0.03)	Z = 0.14 (*P* = 0.89)	
		Leakage	8	1163	0.94 [0.70, 1.26]	I^2^ = 29% (*P* = 0.20)	Z = 0.43 (*P* = 0.67)	
		Cholangitis	3	644	2.68 [0.74, 9.64]	I^2^ = 0% (*P* = 0.82)	Z = 1.51 (*P* = 0.13)	
External stent		Stricture	9	1074	0.76 [0.54, 1.07]	I^2^ = 0% (*P* = 0.67)	Z = 1.59 (*P* = 0.11)	
		Leakage	7	877	0.87 [0.45, 1.65]	I^2^ = 49% (*P* = 0.07)	Z = 0.43 (*P* = 0.66)	
		Cholangitis	2	151	1.25 [0.89, 1.76]	I^2^ = 0% (*P* = 0.64)	Z = 1.30 (*P* = 0.19)	
The use of stent in	LDLT	Stricture	5	913	0.80 [0.55, 1.16]	I^2^ = 24% (*P* = 0.26)	Z = 1.20 (*P* = 0.23)	
		Leakage	4	791	1.03 [0.37, 2.83]	I^2^ = 74% (*P* = 0.010)	Z = 0.06 (*P* = 0.95)	
		Cholangitis	0	0	N/A			
	DDLT	Stricture	10	1595	1.02 [0.67, 1.55]	I^2^ = 42% (*P* = 0.08)	Z = 0.10 (*P* = 0.92)	None
		Leakage	8	873	0.82 [0.60, 1.12]	I^2^ = 0% (*P* = 0.66)	Z = 1.25 (*P* = 0.21)	
		Cholangitis	4	695	2.02 [0.86, 4.75]	I^2^ = 0% (*P* = 0.87)	Z = 1.62 (*P* = 0.10)	
	Pediatric	Stricture	4	586	0.71 [0.44, 1.13]	I^2^ = 0% (*P* = 0.47)	Z = 1.46 (*P* = 0.14)	
		Leakage	3	511	1.30 [0.79, 2.13]	I^2^ = 30% (*P* = 0.24)	Z = 1.02 (*P* = 0.31)	
		Cholangitis	1	99	N/A			
	Adult	Stricture	12	2021	0.95 [0.64, 1.42]	I^2^ = 54% (*P* = 0.01)	Z = 0.25 (*P* = 0.80)	None
		Leakage	10	1252	0.83 [0.63, 1.11]	I^2^ = 36% (*P* = 0.12)	Z = 1.24 (*P* = 0.22)	
		Cholangitis	4	695	2.02 [0.86, 4.75]	I^2^ = 0% (*P* = 0.87)	Z = 1.62 (*P* = 0.10)	
	RYHJ	Stricture	3	467	1.06 [0.61, 1.85]	I^2^ = 0% (*P* = 0.50)	Z = 0.21 (*P* = 0.83)	
		Leakage	3	467	1.47 [0.92, 2.35]	I^2^ = 0% (*P* = 0.37)	Z = 1.60 (*P* = 0.11)	
		Cholangitis	1	52	N/A			
	DD anastomosis	Stricture	6	886	0.74 [0.41, 1.35]	I^2^ = 43% (*P* = 0.12)	Z = 0.98 (*P* = 0.33)	None
		Leakage	4	229	0.94 [0.23, 3.89]	I^2^ = 0% (*P* = 0.51)	Z = 0.08 (*P* = 0.94)	
		Cholangitis	3	643	2.68 [0.74, 9.66]	I^2^ = 0% (*P* = 0.82)	Z = 1.51 (*P* = 0.13)	

### Internal Stent

**Fig. 3:**
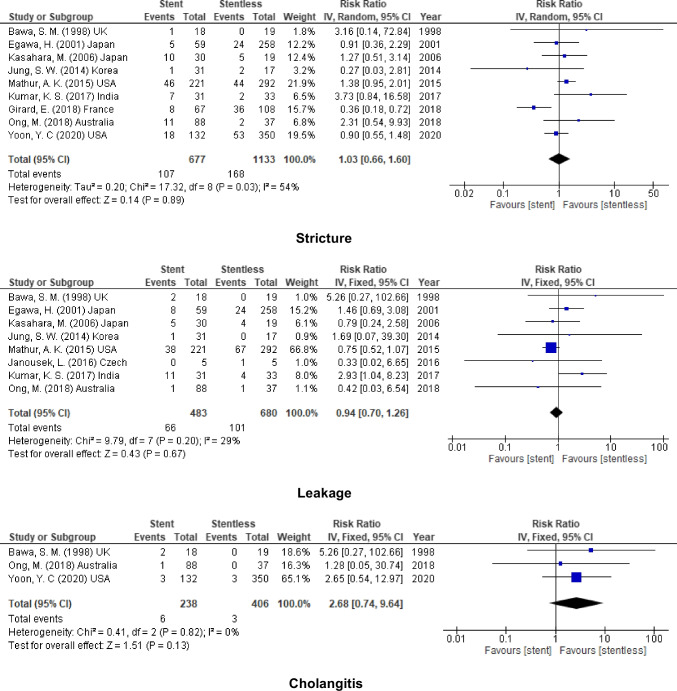
Biliary complication for internal stent vs. stentless

On the one hand, nine of the included studies used internal stent in biliary anastomosis in comparison to biliary anastomosis without stent. Nine studies (1810 participants) reported the incidence of biliary stricture, 8 studies (1163 participants) assessed the rate of leakage, and 3 studies (644 participants) calculated the incidence of cholangitis. The pooled results from these studies showed no added benefit on using internal stent in biliary anastomosis as the same rate of complication could be detected for the two groups. (stricture, RR = 1.03, 95% CI = 0.66–1.60, *P* = 0.89; *I*^2^ = 54%), (leakage, RR = 0.94, 5% CI = 0.70–1.26, *P* = 0.67; *I*^2^ = 29%), and (cholangitis, RR = 2.68, 95% CI = 0.74–9.64, *P* = 0.13; *I*^2^ = 0%) (Fig. 3).

### External Stent

**Fig. 4:**
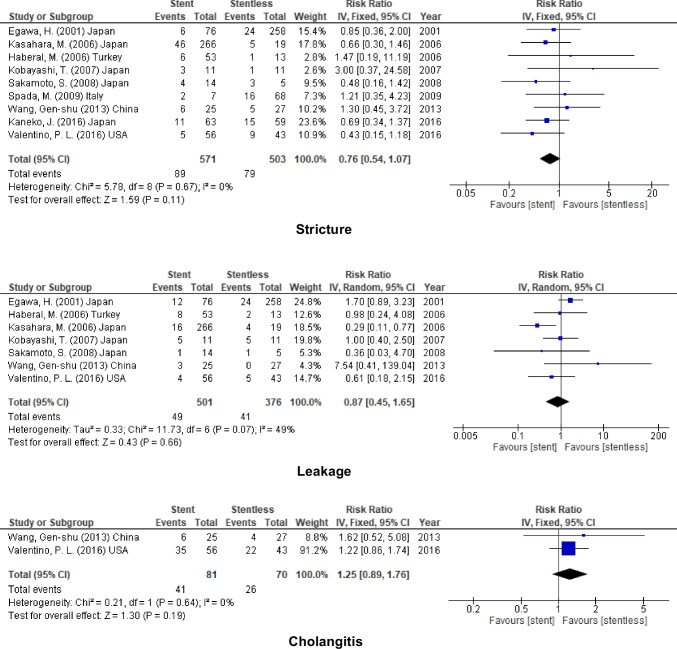
biliary complication for external stent vs stentless

On the other hand, external stent was used in six of the included studies. While the incidence of stricture was reported in the 9 studies (1074 participants) and leakage reported by 7 studies (877 participants), only two of the included studies (151 participants) calculated the rate of post-operative cholangitis. Similar to internal stent, no decrease in the incidence of post-operative complications after using external stent. (stricture, RR = 0.76, 95% CI = 0.54–1.07, *P* = 0.11; *I*^2^ = 0%), (leakage, RR = 0.87, 95% CI = 0.45–1.65, *P* = 0.66; *I*^2^ = 49%), and (cholangitis, RR = 1.25, 95% CI = 0.89–1.76, *P* = 0.19; *I*^2^ = 0%) (Fig. 4).

### Subgroup Analysis

To investigate the effect of using stents versus no stent in different modalities of liver transplantation (LDLT and DDLT), different age groups (adult and pediatric liver transplantation) and various types of reconstructions (duct to duct anastomosis and hepaticojejunostomy).

### Type of the Graft

Turning to the type of the graft, 3 of the included studies used grafts from living donors, 10 studies used grafts from deceased donors, and only one study used both types.

### LDLT

**Fig. 5:**
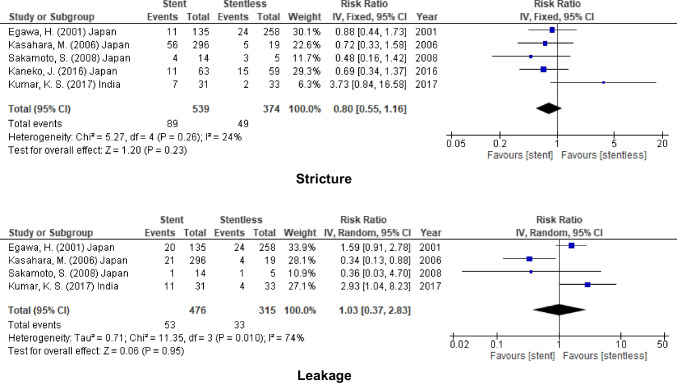
Biliary complication for stent (internal or external) vs. stentless in LDLT

According to 5 of the included studies (913 patients) reported the incidence of stricture and 4 studies (791 participants) reported that for leakage, no significant decrease in the incidence of biliary complications after using biliary stent (Stricture, RR = 0.80, 95% CI = 0.55–1.16, *P* = 0.23, *I*^2^ = 24%) and (leakage, RR = 1.03, 95% CI = 0.37–2.83, *P* = 0.95; *I*^2^ = 74%) (Fig. 5).

### DDLT

**Fig. 6:**
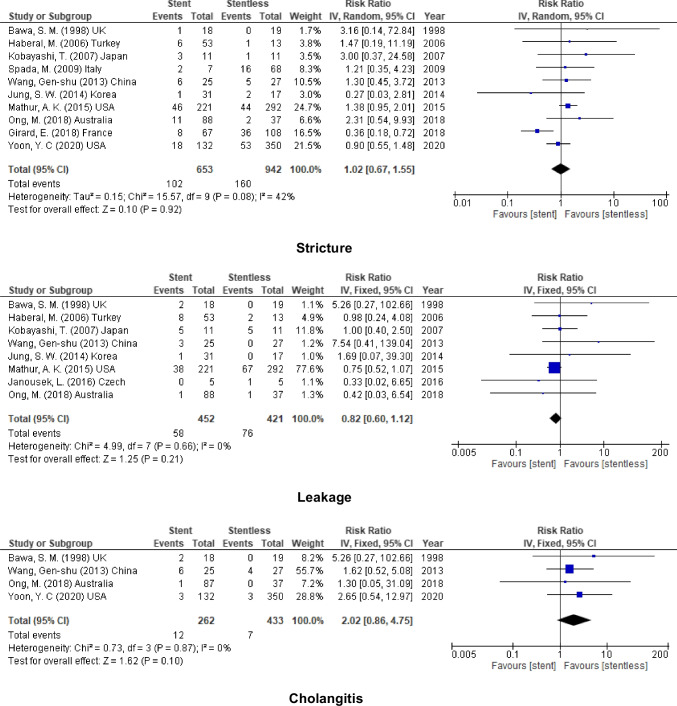
Biliary complication for stent (internal or external) vs. stentless in DDLT

In addition to that, for DDLT, the incidence of biliary complications was similar after using stent in biliary anastomosis (stricture, RR = 1.02, 95% CI = 0.67–1.55, *P* = 0.92; *I*^2^ = 42%), (leakage, RR = 0.82, 95% CI = 0.60–1.12, *P* = 0.21; *I*^2^ = 0%), and (cholangitis, RR = 2.02, 95% CI = 0.86–4.75, *P* = 0.10; *I*^2^ = 0%) (Fig. 6).

### Age of the Recipient

Regarding the age of the recipient, the transplantation in 13 (2031 participants) of the included studies was for adult recipient and in four studies, only (586 patients) transplantation was for pediatrics.

### Adult

**Fig. 7:**
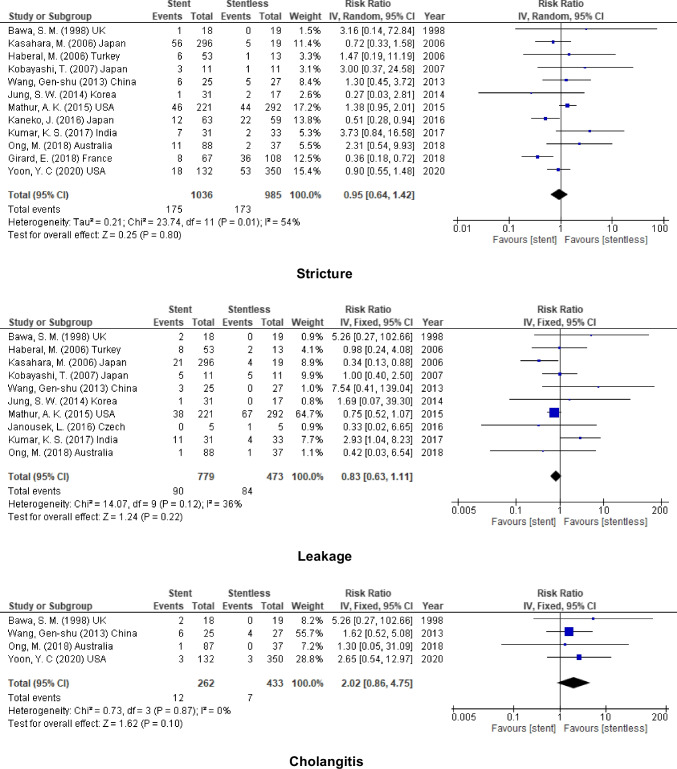
Biliary complication for stent (internal or external) vs. stentless in adult recipient

On the one hand, no significant decrease in the biliary complications after using stent in adult liver transplantation (stricture, RR = 0.95, 95% CI = 0.64–1.42, *P* = 0.80; *I*^2^ = 54%), (leakage, RR = 0.83, 95% CI = 0.63–1.11, *P* = 0.22; *I*^2^ = 36%), and (cholangitis, RR = 2.02, 95% CI = 0.86–4.75, *P* = 0.10; *I*^2^ = 0%) (Fig. 7).

### Pediatric

**Fig. 8:**
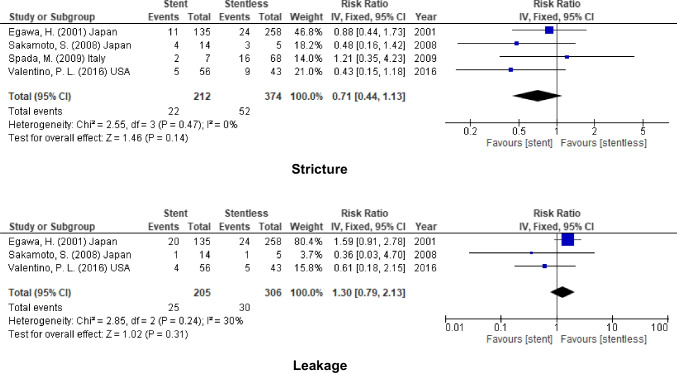
Biliary complication for stent (internal or external) vs. stentless in pediatric recipient

In addition to that, the incidence of biliary stricture and leakage did not decrease by using biliary stent in pediatric liver transplantation (stricture, RR = 0.71, 95% CI = 0.44–1.13, *P* = 0.14; *I*^2^ = 0%) and (leakage, RR = 1.30, 95% CI = 0.79–2.13, P = 0.31; *I*^2^ = 30%) (Fig. 8).

### Type of Biliary Anastomosis

Turing to the type of biliary anastomosis, 6 studies (886 patients) used duct to duct anastomosis, 3 studies (643 patients) used Roux-en-Y hepaticojejunostomy, 5 studies used both modalities, and 3 of the included studies did not specify the type of anastomosis.

### Duct to Duct Anastomosis

**Fig. 9:**
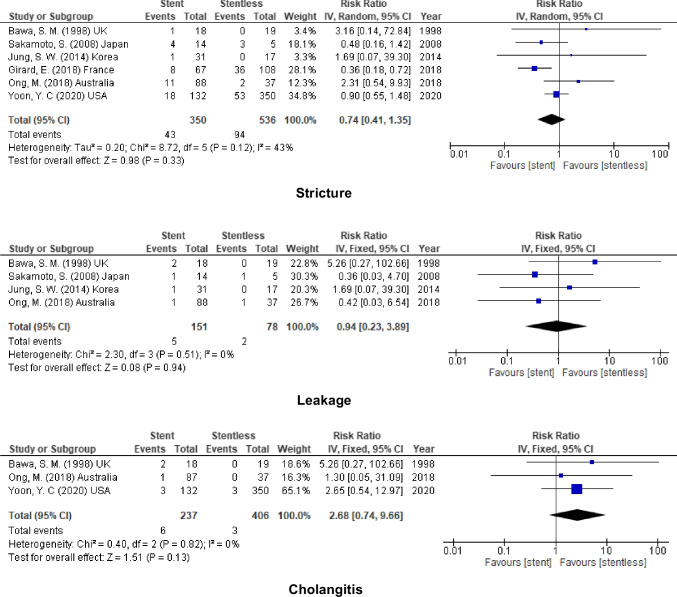
Biliary complication for stent (internal or external) vs. stentless in D-D anastomosis

No significant decrease in the incidence of biliary complications after using stent in duct to duct anastomosis (stricture, RR = 0.74 95% CI = 0.41–1.35, *P* = 0.33; *I*^2^ = 43%), (leakage, RR = 0.94, 95% CI = 0.23–3.89, *P* = 0.94; *I*^2^ = 0%), and (cholangitis, RR = 2.68, 95% CI = 0.74–9.66, *P* = 0.13; *I*^2^ = 0%) (Fig. 9).

### Hepaticojejunostomy

**Fig. 10:**
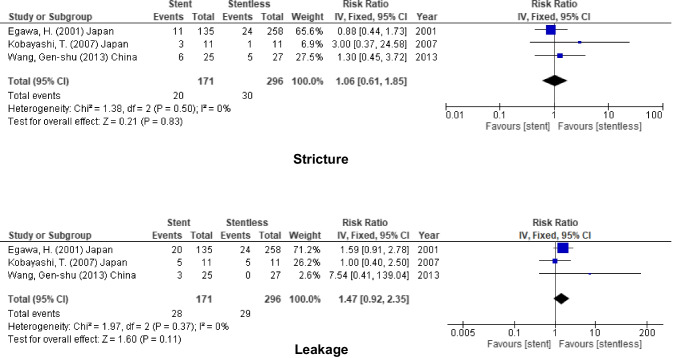
Biliary complication for stent (internal or external) vs. stentless in RYHJ anastomosis

In addition to that, similar to duct to duct anastomosis, no added benefit on using stent in biliary anastomosis (stricture, RR = 1.06, 95% CI = 0.61–1.85, *P* = 0.83; *I*^2^ = 0%), and (leakage, RR = 1.47, 95% CI = 0.92–2.35, *P* = 0.11; *I*^2^ = 0%) (Fig. 10).

#### Publication Bias Assessment

There was no evidence of publication bias. The funnel plot analysis demonstrated a symmetrical appearance, and the *P* values were greater than 0.05 for all comparisons according to the Begg-Mazumdar test and eggers test.

## Discussion

Biliary complications post-transplantation can cause a remarkable decline in the incidence of patient and graft survival.^26^ Forty percent of these complications are from bile duct strictures. Anastomotic biliary stricture account for 80% of all strictures, and non-anastomotic stricture account for 20%.^27^ The majority of these patients will need an average of one sessions of ERCP every 3 months, with stenting and dilation for 1–2 years ^2^. Moreover, the incidence of bile leaks that occur mainly from the anastomotic site range from 2 to 25% post-transplant;^2,28^ and they usually require on average two ERCP sessions.^29^ It is noteworthy to mention that bile leak is a risk factor for strictures and vice versa.^30^ Biliary reconstruction remains the Achilles’ heel of liver transplantation.^31^

According to Ikegami et al., to avoid these complications, the anastomosis should be tension-free, not too long or short, with good blood supply, accurate understanding, and planning of biliary anatomic variation.^32^ In addition to that, many centers have used trans anastomotic biliary stent (internal or external stent) as a way to reduce the incidence of biliary complications. Moreover, the T-tube was previously used to provide an easy access to the biliary system for cholangiography, to allow monitoring of the quality and output of bile and for decompression of biliary system. However, on the one hand, a recent meta-analysis consisting of 1027 patients illustrated that those without a T tube had a decreased incidence of cholangitis and peritonitis with overall decreased rate of biliary complications.^33^ In addition to that, according to Zhoa et al., the recent studies failed to support the routine use of T-tube in adult liver transplantation.^34^

On the other hand, the routine use of biliary stents as a way to decrease the incidence of biliary complications is still controversial. According to a pilot study conducted by Barkun et al., the use of trans-anastomotic biliary stents for duct-to-duct anastomosis significantly decrease the rate of biliary complications when compared to biliary anastomosis without stenting.^5^ Moreover, as reported by Welling et al., the use of biliary stent appeared to be protective against bile leak.^6^ Bawa et al. demonstrated a high rate of biliary complications particularly associated with the use of endobiliary stents and they argued that choledochocholedochostomy without stenting was an efficient and a safe technique for biliary reconstruction.^12^ Mathur et al. also demonstrated that stenting using internal feeding tubes was associated with greater morbidity.^10^ Moreover, Stented patients had a higher risk of needing multiple ERCPs and endoscopic procedures in the first 90 days after transplantation. Verran et al. and Alsharabi et al. also found that internal biliary drainage was not required in liver transplantation.^7,35^ In this meta-analysis, the pooled results from the included studies showed no significant difference in the incidence of post-operative biliary complications between the two groups.

A wide variety of trans anastomotic biliary stents have been used since Terrier reported the use of an internal stent for the first time in 1890.^36^ As previously mentioned, the T-tube that was introduced in 1912 by Kehr as a method for stenting the common bile duct after choledochotomy and providing external drainage, is associated with an increase in the incidence of biliary complications.^37^ On the one hand, according to Jung et al.,^20^ the use of plastic internal stent in biliary reconstructions is associated with a significant decrease in the incidence of biliary complications and Janousek et al. reported that using bio-absorbable stent could also decrease the complication rate. However, according to our study, no added benefit on using internal biliary stent in reconstruction. On the other hand, the use of the external stent that was first described in 1902 by Robson ^38^ after choledochotomy as a stent and as a method for external drainage, failed to decrease the incidence of biliary complications after liver transplantation according to the pooled results of the included studies.

LDLT is an excellent alternative to DDLT especially in the areas with low deceased organ availability.^39^ However, the incidence of biliary complications in LDLT is 2–3 times higher than that in DDLT and as reported by Sharma et al., the rate of biliary complication ranges from 5–15% after deceased donor liver transplantation and 28–32% after living donor liver transplantation.^40^ This could be attributed to the fact that LDLT anastomoses are made between multiple small peripheral bile ducts. ^41^ According to some studies, the surgical experience is associated with a dramatic decrease in the incidence of these complications after LDLT.^42,43^ Other studies reported that the use of trans-anastomotic biliary stent in LDLT can decrease the incidence of post-transplant biliary complications^22^ and according to Croome KP et al., there is a role of leaving a stent for early diagnosis of ischemic cholangiopathy in donation after circulatory death (DCD) livers.^44^ However, according to the pooled results of the included studies, no significant decrease in the biliary complications after the routine use of stent in either LDLT or DDLT.

Since the first report of liver transplantation in 1963, the rate of biliary complications has not changed a lot, with the overall incidence in pediatric LT still ranging from 10 to 35%.^11,43^ Despite the routine use of biliary stent in pediatric liver transplantation in some centers to decrease the biliary complication, no enough studies could be found to support that. In addition to that, according to a survey including surgeons from the highest performing pediatric liver transplantation centers in north America, the routine use of t-tubes in biliary reconstruction should be avoided.^45^ However, according to Sakamoto S. et al., a trans-anastomotic biliary stent should be used in D2D biliary reconstruction in pediatric LDLT to decrease the incidence of biliary complication.^17^ The pooled results of the included studies showed that no added benefit in the routine use of biliary stent in pediatric or adult liver transplantation.

Since 1870 when the first attempt of cholecystoenterostomy was done by Alexandervon Winiwarter, a wide variety of techniques for biliary reconstruction have been described.^46^ However, the two most common forms of biliary reconstruction in liver transplantation are choledochocholedochostomy (CC, duct-to-duct anastomosis) and Roux-en-Y Hepaticojejunostomy (RYHJ, connection of the bile duct to a portion of jejunum). The choice of the type biliary reconstruction depends on surgeon decision as no clear-cut guideline on the best type of biliary reconstruction. It is determined by numerous factors, including the size of recipient and donor bile ducts, underlying liver pathology, and previous transplantation or previous biliary surgery. According to some studies, the incidence of strictures of all types are more prevalent with Roux-en-Y than choledochojejunostomy.^47^ However, according to a meta-analysis comparing duct-to-duct versus Roux-en-Y biliary reconstruction following liver transplantation for primary sclerosing cholangitis, both techniques have similar rate of biliary stricture but RYHJ has a higher risk of cholangitis.^48^ Duct-to-duct anastomosis is the most common biliary reconstruction procedure performed during OLT. This is because, firstly, duct-to-duct anastomosis is faster and easier than RYHJ. Secondly, duct-to-duct anastomosis does not require intestinal manipulation and entero-enteric anastomosis. Thirdly, this type of anastomosis enables easier endoscopic access postoperatively. Lastly, it decreases the incidence of ascending cholangitis theoretically by preservation of the sphincter of Oddi and no reflux of enteric content in the biliary tree.^14,49,50^ Regarding the use of biliary stent, according to Girard E. et al.^24^ and Sakamoto S et al.,^17^ the use of trans-anastomotic biliary stent in duct-to-duct anastomosis is associated with a remarkable decrease in the incidence of biliary complication. In addition to that, Egawa H. et al.^13^ reported that the use of biliary stent can decrease the rate of postoperative leakage and stricture in RYHJ. However, according to our study, no added benefit on the routine use of biliary stent in both modalities as regard biliary complication.

Best of our knowledge, it is the first meta-analysis to compare biliary complications after using trans-anastomotic biliary stent versus no stent in liver transplantation and subgroup analysis was done to detect the effect of using stent in different modalities of liver transplantation (LDLT and DDLT), different age groups (adult and pediatric liver transplantation), and various types of reconstructions (duct to duct anastomosis and hepaticojejunostomy). In addition to that, all the studies comparing between the two groups were included in our study.

However, we have to acknowledge some limitations in our study. Firstly, not all the included studies were randomized controlled trials. Secondly, the existence of significant heterogeneity in some outcomes could not be explained well enough by subgroup analysis. Moreover, none of the included studies compared the acute rejection or the graft loss secondary to biliary complications between the two groups. In addition to that, most of the studies were conducted in Asia and only few of them were conducted in North America and Europe. Lastly, more studies are needed to compare the use of stent versus stentless in pediatric liver transplantation and RYHJ. Moreover, only one study was found comparing the use bio-absorbable stent vs. stent less in biliary reconstruction.

## Conclusion

No added benefit on the routine use of endobiliary stent in liver transplantation including living donor liver transplant (LDLT), deceased donor liver transplant (DDLT), Roux-en-Y hepaticojejunostomy (RYHJ) and duct-to-duct anastomosis, pediatric and adult liver transplantation. However, stented patients are at higher risk of needing multiple ERCPs.
